# Patient-Reported Outcome Measures following Coblation Nucleoplasty for Cervical Discogenic Dizziness

**DOI:** 10.3390/jcm12134413

**Published:** 2023-06-30

**Authors:** Yongchao Li, Bing Wu, Mao Li, Xiaodong Pang, Liang Yang, Chen Dai, Baogan Peng

**Affiliations:** 1 Department of Orthopedics, The Third Medical Centre of Chinese PLA General Hospital, Beijing 100039, China; liyongchaospine@163.com (Y.L.); kevin1126@126.com (B.W.); wjgkpxd@163.com (X.P.); dcanb123@163.com (C.D.); 2 Department of Surgery, Peking University Hospital, Beijing 100034, China; em66666@pku.edu.cn; 3 Department of Orthopeadics, Featured Medical Center of Chinese People’s Armed Police Forces, Tianjin 300162, China; yangliang_spine@163.com

**Keywords:** cervical discogenic dizziness, cervicogenic dizziness, neck pain, coblation nucleoplasty, patient-reported outcome measures

## Abstract

Background: There is little research in the literature comparing the efficacy of coblation nucleoplasty with conservative treatment in the treatment of cervical discogenic dizziness and reporting the achieved rate of minimal clinically important differences (MCID) and patient acceptable symptom state (PASS) after surgery. This retrospective study aims to explore the patient-reported outcome measures (PROM) following coblation nucleoplasty for cervical discogenic dizziness and to compare the therapeutic effect of coblation nucleoplasty with prolonged conservative treatment. Methods: Sixty-one patients with cervical discogenic dizziness and a positive intradiscal diagnostic test eligible for single-level cervical coblation nucleoplasty were included in the study. Among these 61 patients, 40 patients underwent cervical coblation nucleoplasty, while the remaining 21 patients refused surgery and received continued conservative treatment. The primary PROMs were the intensity and frequency of dizziness and secondary PROMs were related to the neck disability index (NDI) and visual analog scale (VAS) for neck pain (VAS-neck) during a 12-month follow-up period. Moreover, the achieved rate of MCID and PASS in both groups was assessed 12 months after surgery. Results: Dizziness intensity, dizziness frequency, VAS-neck score, and NDI score were significantly improved from the baseline at all follow-up time points in both treatment groups, except for showing no significant improvement in dizziness frequency in the conservative treatment group at 6 and 12 months after surgery. However, at each follow-up time point, the above indexes were lower in the surgery group than in the conservative treatment group. In addition, the achieved rates for PASS and MCID in all indexes in the surgery group were significantly higher than those in the conservative treatment group at 12 months after surgery. Conclusions: Cervical coblation nucleoplasty significantly improved the intensity and frequency of dizziness, neck pain, and NDI in patients with cervical discogenic dizziness, and the results were superior to those from prolonged conservative treatment. Meanwhile, cervical coblation nucleoplasty is a good choice for patients with chronic neck pain and refractory cervical discogenic dizziness who have not demonstrated the indications for open surgery and have not responded well to conservative treatment.

## 1. Introduction

Dizziness is one of the most common reasons that adults seek medical care [1]. Takahashi et al. investigated the etiology of 1000 dizziness patients in outpatient clinics and found that cervicogenic dizziness accounted for 89% of all cases [2]. The cervical spine has highly developed proprioceptors, and its afferent information is integrated with that of the visual and vestibular systems in the central nervous system to maintain the coordinated movement of the head, eyes, and neck [1,3]. When degenerative disc diseases occur, abnormal cervical proprioception will be afferent, resulting in a mismatch or conflict with the afferent information provided by the visual and vestibular systems; this will interfere with the orientation of the head and neck in space, and will eventually lead to proprioceptive cervicogenic dizziness [1]. Recent studies have found that the incidence of dizziness is 50% in patients with cervical spondylosis [4] but is as high as 65% in patients over 65 years old [5]. Because cervical spondylosis is characterized by cervical disc degeneration, proprioceptive cervicogenic dizziness appears to be associated with disc degeneration [6]. More recently, Yang et al. [7,8] found that a large number of Ruffini corpuscles growing into diseased cervical discs may be associated with cervicogenic dizziness. However, the Ruffini corpuscle is a type I mechanoreceptor that transmits proprioceptive information to the vestibulospinal nucleus. The contribution of the degenerative cervical disc to proprioceptive cervicogenic dizziness can be further determined by the significant improvement of dizziness after intradiscal analgesic block treatment [8,9] and anterior cervical surgery [8,10,11,12,13]. Therefore, we also define the proprioceptive cervicogenic dizziness caused by cervical disc degeneration as cervical discogenic dizziness [6,8].

Cervical discogenic dizziness should initially be managed with non-surgical treatments, which mainly include physical therapy, manual therapy, vestibular rehabilitation, drug therapy, and acupuncture [1]. The condition of the majority of patients can be effectively improved by strict conservative treatment, but a small number of refractory dizziness patients, who have failed to respond to various non-surgical treatments, can be treated by surgery [1]. A large number of clinical studies [8,10,11,12,13], including a multicenter prospective cohort study [10], have shown that open anterior cervical surgery is effective in improving dizziness in patients with cervical radiculopathy and/or myelopathy. However, this procedure carries the risk of multiple complications, such as dysphagia and adjacent disc disease, and is not suitable for patients with mild disc degeneration. With the development of minimally invasive techniques, cervical coblation nucleoplasty can be a complementary procedure that bridges the gap between conservative treatment and open anterior cervical surgery [14]. Many previous studies have reported that cervical coblation nucleoplasty or coblation discoplasty can effectively treat cervical discogenic dizziness [9,14,15,16]. However, there is little research in the literature comparing the therapeutic effect of coblation nucleoplasty with conservative treatment and reporting the achieved rates of postoperative minimal clinically important differences (MCID) and patient acceptable symptom state (PASS). This study offers a retrospective review of prospectively collected data to explore patient-reported outcome measures following coblation nucleoplasty for cervical discogenic dizziness. At the same time, we compare the therapeutic effect of coblation nucleoplasty with that of prolonged conservative therapy.

## 2. Materials and Methods

### 2.1. Patients

From August 2018 to August 2021, a total of 102 patients with cervical discogenic dizziness who had a positive cervical intradiscal diagnostic test were retrospectively analyzed. Among them, 12 patients underwent non-single-level cervical intradiscal diagnostic tests, 3 patients had incomplete data, 5 patients had cervical trauma, 2 patients had prior cervical surgery, and 19 patients were not suitable for cervical coblation nucleoplasty. Finally, 61 patients eligible for single-level cervical coblation nucleoplasty were included in the study. All patients met the inclusion and exclusion criteria and experienced temporary relief of more than 60% in neck pain and dizziness after an injection of 0.3–0.5 mL bupivacaine into the suspected cervical disc (considered a positive cervical intradiscal diagnostic test). Among these 61 patients, 40 patients underwent cervical coblation nucleoplasty, while the remaining 21 patients refused surgery and received continued conservative treatment. This retrospective review of prospectively collected data was approved by the Ethics Review Committee of our hospital and followed procedures that were in accordance with the Declaration of Helsinki. Informed written consent was given by all patients.

The inclusion criteria for cervical coblation nucleoplasty were as follows: (1) the patient met the diagnostic criteria of cervical discogenic dizziness referred to in our previous review (Table 1) [1]; (2) the patient had chronic or persistent neck pain and intractable dizziness (with a score of 40 or greater on the 100-millimeter visual analog scale) that did not respond well to various forms of conservative treatments for at least 6 months; (3) cervical plain radiographs and magnetic resonance imaging of the patient showed no narrowing of the intervertebral space and mild cervical disc degeneration (Pfirrmann grades Ⅰ–Ⅲ) [17] without cervical nerve root or spinal cord compression.

Patients were excluded if they met one of the following criteria: (1) they had a history of cervical trauma (including whiplash injury) or surgery; (2) they suffered from neurologic diseases, cardiovascular diseases (including atherosclerosis, thickened carotid intima or carotid plaque, or uncontrolled hypertension), vestibular diseases (including benign positional proximal vertigo and Ménière’s disease), vision disorders, and any other systematic or severe diseases; (3) they had any demonstrable abnormalities in the congenital or developmental cervical spine; (4) they had cervical instability (displacement of >3 mm and/or rotational change of >11 compared to the adjacent segments) [14]; (5) they had Bow Hunter’s syndrome, migraines, or coagulopathy; (6) they had undergone two or more segments of cervical coblation nucleoplasty; (7) they had not finished a one-year follow-up and presented incomplete data.

### 2.2. Cervical Coblation Nucleoplasty

**Figure 1:**
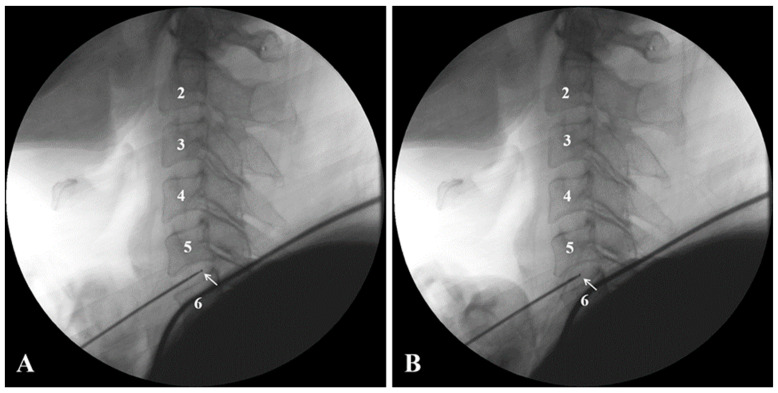
Two different ablation and coagulation areas in the targeted intervertebral disc (C5–C6). The tip of the ablation blade (white arrow) was positioned in the dorsal one-third of the intervertebral disc (**A**) and at the center of the intervertebral disc (**B**).

Cervical coblation nucleoplasty was performed using an anterolateral approach [14,15]. The patient was placed in a supine position with a soft pillow placed at the shoulder to maintain the neck in an extended position and relax the neck muscles. The operator needed to wear lead clothing to reduce radiation exposure. Under C-arm fluoroscopy, the position of the target intervertebral space on the body surface was located and the puncture point was marked. After routine disinfection, 0.5% lidocaine was used for local infiltration anesthesia of the puncture site and prevertebral fascia. At the medial margin of the right sternocleidomastoid muscle, the clinician touched the common carotid artery pulsation. Between the common carotid artery sheath and the tracheoesophagus (visceral sheath), the fingertips of the indicator finger and the middle finger of the clinician were pressed to the anterior midline of the cervical spine, and the target cervical intervertebral disc was entered between the two fingers with a special puncture needle, under C-arm monitoring. During such a procedure, repeated punctures that are not monitored by the C-arm should be avoided since they carry the risk of esophageal injury and intervertebral disc infection. Subsequently, standard cervical anterior-lateral radiographs were taken to confirm that the needle tip was located on the dorsal one-third of the intervertebral disc. Then, the needle core was pulled out and the cervical disc ablation blade (Perc-DC SpineWand) was inserted so that it reached the dorsal one-third of the disc and did not exceed the posterior edge of the vertebral body, under C-arm monitoring (Figure 1A). The power supply was connected to the ArthroCare System 2000 radiofrequency machine (ArthroCare, Sunnyvale, CA, USA). After confirming that the tip was in a secure position and without abnormal spasm or movement following 0.5–1 s of coagulation stimuli, three ablation and coagulation cycles (10 s each) were performed, rotating the wand 360 degrees each time. Subsequently, the second round of radiofrequency and ablation was performed in the same manner by withdrawing the ablation blade tip by approximately 2–3 mm to the center of the intervertebral disc (Figure 1B). After surgery, the skin puncture site was covered with a bandage, and cervical collar protection was used for 2 weeks. 

The surgical levels involved range from C3–C4 to C6–C7. The distribution of surgical segments in the surgical group was similar to that in the conservative group (Table 2). In the conservative treatment group, intervention strategies include physical therapy, manual therapy, temporary cervical collar fixation, and the use of nonsteroidal anti-inflammatory drugs and muscle relaxants.

### 2.3. Patient-Reported Outcome Measures (PROMs)

This study used PROMs to evaluate the treatment benefits. PROMs refer to any reports received directly from patients regarding their health status, without the need for evaluation by clinical doctors or anyone else. The primary PROMs comprised the intensity and frequency of dizziness. The intensity of dizziness was measured with a 100-millimeter visual analog scale (VAS) and the frequency of dizziness was measured on a six-point categorical rating scale (0 = no dizziness, 1 = dizziness < once per month, 2 = 1–4 episodes per month, 3 = 1–4 episodes per week, 4 = dizziness once daily, 5 = dizziness > once a day or constantly) [8,10,18]. The secondary PROMs comprised the neck disability index (NDI) score and 100-millimeter VAS score for neck pain (VAS-neck). The NDI is a disability survey consisting of 10 questions, each ranging from 0 to 5 points, with a maximum score of 50 points, reported as a percentage out of 100. A higher score indicates a higher degree of disability [8]. The PROMs were assessed at baseline (preoperative) and at 1, 3, 6, and 12 months. Delta scores (Δ) were calculated by subtracting the preoperative score from the 12-month postoperative score. Patients who achieved minimal clinically important differences (MCID) by 12 months after surgery were reported according to previously defined criteria: −15 (30%) for ΔNDI and −25 for ΔVAS-neck and ΔVAS-dizziness [19]. In addition, the NDI, VAS-dizziness, and VAS-neck rates that reached a patient acceptable symptom state (PASS) at 12 months after surgery were also recorded. According to the threshold values used in previous reports [20,21,22], patients with an NDI of ≤17 (34%), VAS-dizziness of ≤30, and VAS-neck of ≤30 consider their postoperative symptom state to be acceptable.

### 2.4. Statistical Analysis

When comparing the baseline data and the MCID and PASS achieving rates between the two groups, chi-square analysis was used for categorical data, represented by count (percentage), while an independent sample *t*-test was used for the normally distributed continuous data, represented by the mean (standard deviation (SD)). VAS-dizziness, VAS-neck, NDI, and dizziness frequency values were compared between the groups at different time points in the follow-up period using a two-way repeated-measure ANOVA. Prism 8 (GraphPad) was used for the statistical analyses. Statistical significance for the analyses in this study was set at a *p*-value of <0.05. 

## 3. Results

### 3.1. General Characteristics

There were no statistically significant differences in age, sex, disease level, and baseline PROMs between the two groups (Table 2). The average operation time of the surgery group was 28.9 ± 12.1 min. The operations were successfully completed in all patients without neurological complications, vascular injury, or other serious adverse reactions during and after the operation. However, four patients developed ecchymosis at the needle puncture site, temporary neck stiffness, and pain behind the ear after surgery, all of which improved with NSAIDs, muscle relaxants, and physical therapy.

### 3.2. Intensity of Dizziness

**Figure 2:**
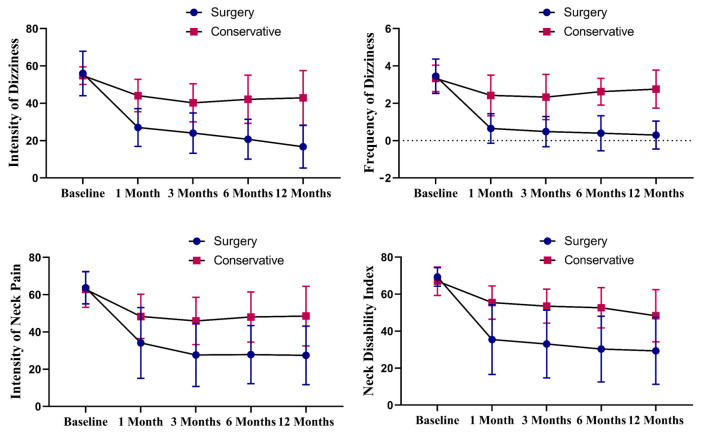
Changes in intensity of dizziness (VAS scores), the frequency of dizziness, the intensity of neck pain (VAS scores), and the neck disability index over time in both groups.

Patients in both treatment groups showed significant improvement in the intensity of dizziness from baseline at all follow-up time points (Table 3 and Figure 2). However, at each follow-up time point, dizziness intensity was lower in the surgery group than in the conservative treatment group (Table 3 and Figure 2). The surgery group achieved MCID VAS-dizziness and PASS VAS-dizziness at significantly higher rates than the conservative group ((MCID: 82.5 versus 28.6%; *p* < 0.001) (PASS: 77.5 versus 23.8%; *p* < 0.001)) (Table 4). 

### 3.3. Frequency of Dizziness

Patients in the surgery group showed significant improvement in terms of the frequency of dizziness from baseline at all follow-up time points, but the improvement was not significant in the conservative group (*p* = 0.050 at 6 months and *p* = 0.196 at 12 months), except in the first and third months after treatment (*p* = 0.002 at 1 month and *p* = 0.003 at 3 months; Table 3 and Figure 2). However, the frequency of dizziness was significantly lower in the surgery group than in the conservative group at each follow-up time point (Table 3 and Figure 2).

### 3.4. Intensity of Neck Pain

After treatment, both groups of patients showed a recovery trend from the first month to the twelfth month after surgery and showed significant improvement from baseline at all follow-up time points (Table 3 and Figure 2). Furthermore, the intensity of neck pain in the surgery group was lower than that in the conservative group at each time point (Table 3 and Figure 2). Patients in the conservative group achieved MCID for VAS-neck at a significantly lower rate than the surgery group (33.3% versus 82.5%; *p* < 0.001; Table 4). In addition, patients in the surgery group achieved PASS for VAS-neck at a significantly higher rate than the conservative group (75.0% versus 23.8%; *p* < 0.001; Table 4).

### 3.5. Neck Disability Index

Both groups showed significant improvement in terms of NDI scores from baseline at all time points after treatment (Table 3 and Figure 2). In addition, at each follow-up time point, the NDI scores were lower in the surgery group than in the conservative group (Table 3 and Figure 2). The rate of achieving MCID for the NDI in the conservative group was significantly lower than that in the surgery group (38.1% versus 75.0%; *p* = 0.005; Table 4). Furthermore, the rate of achieving PASS for the NDI in the surgery group was significantly higher than that in the conservative group (77.5% versus 28.6%; *p* < 0.001; Table 4). 

## 4. Discussion

To the best of our knowledge, there is little research in the literature as to the efficacy of cervical coblation nucleoplasty compared with prolonged conservative treatment in the treatment of cervical discogenic dizziness, as well as the postoperative attainment rates of PASS and MCID. In the current study, we found that both surgical treatment and conservative treatment can improve the symptoms of dizziness and neck pain. However, neck pain and dizziness relief were significantly better in the surgery group than in the conservative treatment group at each follow-up time point. In addition, the achieved rates of PASS and MCID for all indexes (including VAS-dizziness, dizziness frequency, VAS-neck pain, and NDI) in the surgery group were significantly higher than those in the conservative treatment group at 12 months after surgery, indicating that the overall treatment effect of the surgery group was significantly better than that of the conservative treatment group. 

For most patients with cervical discogenic dizziness, conservative treatments such as manual therapy and physiotherapy are effective. However, a small number of patients with refractory dizziness do not respond to the various conservative treatments and can be treated with surgery on the premise of a clear diagnosis [1]. Numerous studies have shown that open anterior cervical surgery is effective in improving dizziness in patients with radiculopathy and/or myelopathy [10,11,12]. In addition, anterior cervical surgery may also improve neck pain and dizziness in patients with severe cervical disc degeneration [8] or cervical instability [13] but who are without radiculopathy and/or myelopathy. However, open anterior cervical surgery carries the risk of multiple complications, such as dysphagia and adjacent vertebral disease, and is not suitable for mild disc degeneration. In recent years, a large number of studies have reported that cervical coblation nucleoplasty or coblation discoplasty can effectively treat the refractory dizziness associated with mild disc degeneration [9,14,15,16]. In a retrospective outcome study of 74 consecutive patients with a minimum of 1 year of follow-up, 85.1% of patients had good or excellent results one week after cervical coblation nucleoplasty, compared to 75.7% at the last follow-up [15]. Further, in another 6-year follow-up study involving 40 patients with cervical degenerative diseases in whom both neck pain and cervical vertigo were treated by coblation nucleoplasty, the rates of completion in 1-year short-term, 3-year mid-term, and 6-year long-term follow-ups were 100%, 100%, and 85%, respectively. The clinical effective rates were 67.5%, 67.5%, and 52.94% in short-term, mid-term, and long-term follow-ups, respectively [14]. In addition, He et al. [9] found that coblation discoplasty significantly ameliorated the severity and frequency of cervicogenic dizziness, and was superior to that reported for the conservative treatment group. However, this study did not assess post-operative MCID and PASS attainment rates. At the same time, Li et al. [14] and He et al. [9] believe that cervical coblation nucleoplasty or coblation discoplasty can be a complementary procedure that bridges the gap between conservative treatment and open anterior cervical surgery to address cervical degenerative diseases and cervicogenic dizziness. The data from our study also support the proposal that cervical coblation nucleoplasty is a good choice for patients with chronic neck pain and refractory cervical discogenic dizziness who do not meet the indications for open surgery and do not respond well to conservative treatment.

In clinical practice, we found that dizziness was more common in patients who showed simple cervical disc degeneration on imaging but had no disc herniation or spinal cord or nerve compression. The main clinical features of the patients included in this study were mild cervical disc degeneration, along with chronic neck pain and persistent dizziness, that their symptoms, especially refractory dizziness, seriously affected their lives and work, and that they did not respond well to conservative treatment. Subsequently, we performed a cervical intradiscal diagnostic test on each suspected cervical disc to identify the location of symptoms. One day before surgery, the suspected degenerative cervical disc was given an intradiscal diagnostic injection of bupivacaine (0.25%, 0.3–0.5 mL). The diagnostic test is considered positive only if the symptoms of neck pain and dizziness are relieved by more than 60%, indicating that the suspected disc is the culprit, and the further target disc will be treated with cervical coblation nucleoplasty. 

A large number of clinical and immunohistochemical studies have found that intervertebral disc degeneration and the ingrowth of Ruffini corpuscles are one of the causes of proprioceptive cervicogenic dizziness [7,8,10,12,13]. A human cervical intervertebral disc has both free nerve endings and mechanoreceptors (mainly Ruffini corpuscles), but these are only distributed in the outer annulus fibrosus [7,23]. Recently, three immunohistochemical studies have shown that a large number of Ruffini corpuscles and free nerve endings have grown into the inner annulus fibrosus and even the nucleus pulposus in diseased cervical intervertebral discs obtained from patients with cervical spondylosis [7], only cervical disc degeneration [8], or severe chronic neck pain [24]. The main role of Ruffini corpuscles is thought to be to maintain muscle tone and monitor body position, speed, and kinesthesia in conjunction with the information transmitted by muscle spindles, and to play a role in proprioceptive transducer function [10]. Disc degeneration is characterized by elevated inflammatory cytokines that stimulate corpuscles or free nerve endings in the degenerative disc, leading to peripheral sensitization that produces pain and the abnormal release of proprioceptive afferents [6,8]. Abnormal neck proprioceptive input from the Ruffini corpuscles is transmitted to the central nervous system, resulting in a sensory mismatch with the vestibular and visual information, thereby causing dizziness [1,6,7]. However, a degenerative cervical disc does not always cause dizziness, just as a degenerative cervical disc does not always cause neck pain. If the degenerated cervical disc does not have a sufficient number of Ruffini corpuscles and does not have a strong inflammatory response, it may not produce a strong enough proprioceptive afferent impulse [6]. However, preoperative intradiscal analgesic injections can temporarily block Ruffini corpuscles and free nerve endings to achieve temporary pain and dizziness relief, which provides a guarantee of good results from cervical coblation nucleoplasty. In the present study, we selected the inner annulus fibrosus (the dorsal one-third of the disc) and nucleus pulposus (the center of the disc) as therapeutic targets to inactivate the abnormal ingrowth of Ruffini corpuscles and free nerve endings, while inhibiting the inflammatory response in the nucleus pulposus. In a study by Li et al. [14], these two targets were also selected, and a good long-term therapeutic effect was achieved (of more than 6 years). In addition, another study that only selected the nucleus pulposus as the target achieved satisfactory results in 75.7% of patients 1 year after surgery [15]. Recently, in addition to the above two targets, He et al. [9] also selected the edge of the annulus fiber as the target, which also provided results that were consistent with those in the present study. However, there is a risk of damage to the posterior longitudinal ligament and dural sac, as well as a reduction in disc height, when the annulus margin is selected as a target. 

On the other hand, neck pain often causes proprioceptive deficits and neck muscle tension [1,25]. In clinical practice, we have found that patients with nonspecific neck pain report that it is often accompanied by proprioceptive and spatial disorientation. A narrative review by Peng et al. [25] found that one of the main problems for patients with neck pain is that changes in neck proprioception lead to impairments in neck sensorimotor control. Malmström et al. [26] found that acute neck pain induced by a hypertonic saline injection in the splenius capitis muscle on one side could lead to proprioceptive disorders in the neck. The clinical implications of this finding are that neck pain itself has a clear role in proprioception and neck sensorimotor control and subsequently affects spatial orientation. The main function of pain is to prevent further tissue damage. This corresponds to reduced muscle activity in the painful muscles, which shifts from the deep painful muscles to the superficial muscles and, thus, may interfere with normal proprioception [26]. Chronic neck pain has been reported to affect neck muscle size, thickness, strength, accuracy, acuity, endurance, range of motion, and cervical joint position errors [8,25,27]. These changes in the structure and function of the neck muscles can alter proprioceptive discharge, thereby affecting the afferent input and leading to proprioceptive abnormalities [28]. In addition, neck pain that is caused by cervical disc degeneration may increase muscle spindle sensitivity, while neck muscle tension can also induce γ-muscle spindles to produce abnormal neck proprioceptive input [6,29]. Therefore, relieving neck pain may normalize abnormal proprioceptive afference and further reduce the intensity and frequency of dizziness. This study found that at all time points of the postoperative follow-ups, neck pain and NDI scores in the surgery group were significantly lower than those before surgery and were better than those in the conservative treatment group. At the same time, improvements in the postoperative intensity and frequency of dizziness followed a similar trend to changes in neck pain and NDI scores. 

## 5. Limitations

There are some potential limitations to our study that need to be mentioned. First, because the exact pathogenesis of cervical discogenic dizziness is still unclear, the diagnostic criteria are mainly in terms of exclusivity. Therefore, further studies are needed to evaluate in detail the validity and sensitivity of our proposed diagnostic criteria for cervical discogenic dizziness and the cervical intradiscal diagnostic test. Second, although the use of a cervical brace as a conservative treatment for cervical discogenic dizziness is not an up-to-date treatment strategy, the immobilization provided by a cervical brace can help to improve neck pain [30] and, thus, help to reduce dizziness further. In the conservative treatment group, we used a temporary cervical collar fixation, while in the surgical group, we used a conventional cervical brace for protection for 2 weeks after surgery. These differences may interfere with the comparison of results between the two groups. Third, our study only completed one year of follow-up, and further follow-up is needed to assess long-term effects. Finally, this study is a retrospective review of prospective data collection, and selection bias may be present. Further prospective randomized controlled trials may determine the outcome of coblation nucleoplasty surgery more accurately regarding cervical discogenic dizziness and neck pain.

## 6. Conclusions

This study indicates that cervical coblation nucleoplasty significantly improved the intensity and frequency of dizziness, neck pain, and NDI in patients with cervical discogenic dizziness, and was superior to prolonged conservative treatment. Our study further suggests that intervertebral disc degeneration alone, without cervical spinal cord and/or nerve root compression, can cause cervical discogenic dizziness and pain; thus, a cervical intradiscal diagnostic test can provide support for the diagnosis of cervical discogenic dizziness and ensure good results from coblation nucleoplasty for the treatment of this disease.

## Figures and Tables

**Table 1:** Diagnostic criteria for cervical discogenic dizziness.

Diagnostic Criteria:
A. Clinical, laboratory, and/or imaging evidence of degenerative disc diseases that are known to cause dizziness. B. Temporal coincidence of the appearance of or increase in both neck pain and dizziness. C. Evidence demonstrated by at least two of the following: 1. Dizziness has developed in temporal relation to the onset of degenerative disc diseases. 2. Dizziness has significantly improved or been resolved in parallel with an improvement in or resolution of degenerative disc diseases. 3. At least two clinical diagnostic tests (cervical torsion test, cervical joint position error, or posturography) are positive. 4. Dizziness is abolished following the diagnostic blockade of cervical intervertebral discs. D. Exclusion of other possible sources of dizziness, including vestibular, visual, central nervous system or psychosomatic pathologies.

Notes: (1) Abnormal imaging findings of the cervical spine are common in people without dizziness; they are suggestive but are not considered exact etiological evidence. (2) Tumors, fractures, infections, and rheumatoid arthritis of the cervical spine have not been formally validated as causes of dizziness but can be accepted to fulfill criteria A in individual cases.

**Table 2:** Comparison of the demographics of the patients in the two groups at baseline.

Characteristic	Surgery Group (*n* = 40)	Conservative Group (*n* = 21)	*p*-Value
Sex, female, *n* (%)	28 (57.0%)	14 (43.8%)	0.789
Age (yrs), mean (SD)	45.2 (8.6)	44.3 (7.1)	0.685
VAS for dizziness, mean (SD)	56.0 (11.9)	54.8 (4.7)	0.666
Dizziness frequency, mean (SD)	3.5 (0.9)	3.3 (0.7)	0.620
VAS for neck pain, mean (SD)	63.7 (8.6)	62.8 (9.6)	0.734
NDI, mean (SD)	69.3 (5.0)	67.0 (7.7)	0.182
Disease Level, *n* (%)			0.590
C3/4	6 (15.0%)	4 (19.0%)	
C4/5	15 (37.5%)	9 (42.9%)	
C5/6	12 (30.0%)	7 (33.3%)	
C6/7	7 (17.5%)	1 (4.8%)	

Data are mean (SD) or number (%). SD, standard deviation; VAS, visual analog scale; NDI, neck disability index.

**Table 3:** All outcome measures over 12 months for each treatment group.

Measure	Baseline	1 Month	3 Months	6 Months	12 Months
VAS-dizziness, mean (SD)					
Surgery group	56.0 (11.9)	27.0 (10.1) *	24.0 (10.8) *	20.7 (10.7) *	16.7 (11.4) *
Conservative group	54.8 (4.7)	44.1 (8.7) *	40.2 (10.2) *	42.1 (12.9) **	42.9 (14.6) **
*p*-Value	0.666	<0.001	<0.001	<0.001	<0.001
Frequency of Dizziness, mean (SD)					
Surgery group	3.45 (0.92)	0.65 (0.79) *	0.48 (0.81) *	0.40 (0.94) *	0.30 (0.75) *
Conservative group	3.33 (0.71)	2.42 (1.09) **	2.33 (1.21) **	2.62 (0.72)	2.76 (1.02)
*p*-Value	0.620	<0.001	<0.001	<0.001	<0.001
VAS-neck pain, mean (SD)					
Surgery group	63.7 (8.6)	34.1 (19.0) *	27.6 (16.9) *	27.8 (15.6) *	27.4 (15.7) *
Conservative group	62.8 (9.6)	48.3 (11.9) **	45.9 (12.7) *	48.0 (13.5) *	48.5 (16.0) *
*p*-Value	0.734	0.003	<0.001	<0.001	<0.001
NDI, mean (SD)					
Surgery group	69.3 (5.0)	35.4 (18.8) *	33.0 (18.3) *	30.3 (17.8) *	29.3 (18.1) *
Conservative group	67.0 (7.7)	55.4 (9.0) **	53.5 (9.2) **	52.6 (10.9) **	48.3 (14.1) *
*p*-Value	0.182	<0.001	<0.001	<0.001	<0.001

*n* = 40 (surgery group), *n* = 21 (conservative group). SD, standard deviation; VAS, visual analog scale; NDI, neck disability index. Comparison with baseline, * *p* < 0.001, ** *p* < 0.01.

**Table 4:** The rates of NDI, VAS-dizziness, and VAS-neck for achieving the MCID and PASS.

Variables	Surgery Group (*n* = 40)	Conservative Group (*n* = 21)	*p*-Value
MCID NDI			0.005
No	10 (25.0%)	13 (61.9%)	
Yes	30 (75.0%)	8 (38.1%)	
MCID VAS-neck pain			<0.001
No	7 (17.5%)	14 (66.7%)	
Yes	33 (82.5%)	7 (33.3%)	
MCID VAS-dizziness			<0.001
No	7 (17.5%)	15 (71.4%)	
Yes	33 (82.5%)	6 (28.6%)	
PASS NDI			<0.001
No	9 (22.5%)	15 (71.4%)	
Yes	31 (77.5%)	6 (28.6%)	
PASS VAS-neck pain			<0.001
No	10 (25.0%)	16 (76.2%)	
Yes	30 (75.0%)	5 (23.8%)	
PASS VAS-dizziness			<0.001
No	9 (22.5%)	16 (76.2%)	
Yes	31 (77.5%)	5 (23.8%)	

Data are expressed as a count (%). MCID, minimal clinically important differences; PASS, patient acceptable symptom state; NDI, neck disability index; VAS, visual analog scale.

## Data Availability

The data presented in this study are available upon reasonable request from the corresponding author (B.P.).
